# Beyond the Focus: The Piriform-Entorhinal-Dentate Circuit as a Driver of Network Hyperexcitability in Epilepsy

**DOI:** 10.1177/15357597261492556

**Published:** 2026-10-01

**Authors:** Katrina Portelli, Davide Giampiccolo, Gabriele Lignani

**Affiliations:** Research Department of Epilepsy, 61554 Queen Square Institute of Neurology, University College London

## Abstract

**Piriform seizures mediated by the piriform-entorhino-dentate circuit induce brainwide functional reorganization in mice**

Yan Tao, Yuxin Zhao, Wenqi Zhong, Jiajia Zhang, Hongyan Zhu, Xutao Zhu, Zikun Wang, Na Wang, Liqin Yang, Fuqiang Xu, Ruiqi Wu. *PLoS Biol.* 24(2): e3003577 (2026).

Systematic identification of global epileptic reorganization and critical seizure-controlling circuits is essential for comprehending epilepsy pathophysiology and for developing network-guided targeted therapies. The piriform cortex (PC) is a recognized epileptogenic region, but how its hyperactivity reshapes whole-brain dynamics and which specific circuits mediate seizures remains unclear. Through multimodal integration of optogenetics, fMRI, electrophysiology, Ca2 + imaging, neural tracing, and circuit-specific manipulation, we mapped the whole-brain dynamics following optogenetic stimulation of PC and identified the fundamental circuit governing piriform seizures. We observed pronounced generalized seizures in mice via repeated optogenetic stimulation of PC *Vglut1* + neurons. Optogenetic kindling of PCVglut1 induced widespread blood-oxygen-level-dependent (BOLD) signal hyperactivation and resting-state functional connectivity (rsFC) alterations, notably sustained hyper­activation in the lateral entorhinal cortex (Lent) and enhanced PC-Lent rsFC. Chronic elimination of Lent neurons receiving PC projections significantly decreased the Lent-dentate gyrus (DG) rsFC. Disruption of the PC-Lent or Lent-DG circuit effec­tively suppressed PC-stimulation-triggered seizures and brain-wide hyperactivation. Our findings demonstrate the dominant role of the PCVglut1-Lentglut-DG circuit in mediating piriform seizures and driving their resulting brain-wide functional reorganization, offering new insights for targeted epilepsy treatments.

## Commentary

Temporal lobe epilepsy (TLE) is the most common form of focal, drug-resistant epilepsy. Focus-centred resection of the amygdala and hippocampus together with the anterior temporal lobe results in circa 70% of patients becoming seizure free after 1 year. However, only half of these maintain seizure freedom in the long term,^1^ meaning the current surgical procedure is often not a cure. Shifting to a network-based approach may provide lasting seizure control but requires the elucidation of key circuits, nodes, and cell subtypes involved in epileptogenesis and seizure propagation. One key node that has been identified is the piriform cortex (PC).^2^ This is a richly connected region, critical for olfactory processing and, importantly, highly vulnerable to seizures (and therefore termed Area Tempestas). Indeed, the PC has been recognized as an essential epileptogenic zone across species.^2-5^ However, precisely how focal hyperactivity within the PC triggers brain-wide network dysfunction and the specific synaptic circuitry responsible for this propagation have remained elusive.^3,4^

In an attempt to answer these fundamental questions, Tao et al have provided a comprehensive causal investigation of the circuits involved in seizure propagation and suppression in temporal lobe seizures originating from the PC.^3^ Using a multimodal framework that integrates optogenetic functional MRI (ofMRI), electrophysiology, *in vivo* calcium imaging, and viral circuit tracing, the authors identify critical regions downstream of the PC, describe functional changes in activity and connectivity in and between these regions, and narrow down cell subtypes involved.

First, they linked PC atrophy to human TLE using structural human MRI. They show that both left- and right-sided TLE patients suffered a 30-31% reduction in PC volume when compared to healthy controls, underscoring the structural vulnerability of the PC to epileptic pathology.^3^

Transitioning to a mouse model, the authors selectively targeted Vglut1 + excitatory neurons in the anterior PC using optogenetic kindling. This selective stimulation readily induced generalized seizures and epileptiform discharges accompanied by robust calcium signal increases. Importantly, they demonstrated that this seizure propagation depends on synaptic transmission, as blocking pre-synaptic glutamate release from PC neurons using a genetically encoded tetanus toxin completely suppressed the seizures. This suggests that the PC is not only causally involved in temporal lobe seizures, but its expression is mediated by long-range white matter transmission.

The authors therefore focused on identifying the network behind piriform seizures. To capture brain-wide functional changes driven by these seizures, the authors employed high-field (9.4T) ofMRI. Following PC kindling, they observed a rapid increase in blood-oxygen-level-dependent (BOLD) responses across several regions. A small subset of these regions, including the medial prefrontal cortex and the lateral entorhinal cortex (Lent), showed rapid and long-lasting hyperactivation. Seed-based resting-state fMRI further revealed significantly enhanced functional connectivity between the PC and the Lent, post-seizure, which was structurally supported by anterograde monosynaptic tracing showing that the Lent receives the highest number of direct projections from the PC.

This structural and functional evidence led the authors to focus their following investigations on the PC-Lent circuit, through a compelling series of targeted circuit manipulations.^3^ Activating the PC terminals in the Lent was sufficient to evoke generalized seizures in a majority of the tested mice. Conversely, using a dual-virus intersectional approach to selectively silence the PC-targeted Lent neurons, they found that obstructing the PC-Lent pathway significantly decreased seizure incidence, eliminated epileptiform discharges, and abolished the BOLD hyperactivation induced by PC kindling. This establishes the PC-Lent pathway as a main connection for piriform seizure propagation.

Then the authors investigated the dentate gyrus (DG) as the next critical node in this epileptic network. Resting-state functional connectivity analyses, as well as anterograde and retrograde viral tracing methods, allowed for the identification of the PC-Lent-DG circuit. Turning then to seizure propagation across this circuit, multichannel calcium imaging revealed a sequential spread of ictal activity: from the PC, to the Lent, and finally to the DG, with distinct inter-regional delays of 2 to 4 s at each step. Across days, the duration of calcium responses increased for each of the three nodes, suggesting a progressive potentiation of connectivity. Finally, chronic elimination of synaptic transmission between the Lent and the DG significantly reduced the number of PC-induced seizures, demonstrating that the downstream recruitment of the DG is essential for the generalization of these seizures.^3^

These findings carry profound implications for our understanding of TLE circuitry and future therapeutic development. Traditionally, the field has heavily focused on hippocampal output pathways, such as the fornix within the classical Papez circuit, in the spread of TLE seizures. Here, the authors introduce a novel “piriform axis,” ie, the PC-Lent-DG circuit, which serves as a major input pathway transmitting epileptic activity into the hippocampus. The authors suggest that the recurrent excitation within this circuit likely serves to amplify focal seizures into generalized events.

From a clinical perspective, the PC-Lent-DG circuit may represent an important new candidate target. Recent evidence on surgical resection^6^ and laser interstitial thermal therapy^7^ suggests that removal of the PC, rather than the hippocampus, may underlie seizure freedom in human TLE. Still, the variability in seizure outcome after resection or ablations in TLE, as well as evidence that seizure recurrence may accrue over the long term, may suggest focus-based perspectives of TLE do not fully capture this disorder. While Lent-DG disconnection is inherently part of current surgical procedure, Tao et colleagues’ work further suggests that elimination of the anterior PC, via radiofrequency ablation or resection, may represent emerging therapeutic routes for treating TLE. As a result, identification of this pathway in humans and understanding how this impacts TLE is paramount.

These findings also open several important directions for future research. The focus on the anterior PC provides a strong foundation for exploring whether the anterior and posterior subdivisions, which have distinct connectivity and functional profiles,^8^ make different contributions to seizure propagation. Extending the validation of the PC-Lent-DG circuit to chronic acquired TLE models, such as those induced by kainic acid or pilocarpine, will also help establish its broader relevance and determine how disease-related inputs to the PC influence the recruitment of downstream regions. Finally, understanding how this pathway is organized in humans remains crucial to therapeutic translation.

More broadly, this network-level view creates new therapeutic opportunities: advanced gene-based^9^ or neuromodulatory therapies^10^ designed to reduce regional excitability could potentially be adapted to selectively target one or more critical propagation nodes within the PC-Lent-DG circuit. Further investigation of how synaptic and non-synaptic mechanisms interact as seizures evolve will refine this emerging circuit framework.

In conclusion, the study by Tao et al elegantly and robustly delineates the synaptic circuitry driving piriform seizures and identifies a compelling pathway for seizure propagation. By reframing the PC from a discrete epileptogenic focus as a critical node within the PC-Lent-DG axis, this work provides a strong foundation for the development of precise, circuit-based therapeutic strategies for refractory epilepsy.
